# STING mediates experimental osteoarthritis and mechanical allodynia in mouse

**DOI:** 10.1186/s13075-023-03075-x

**Published:** 2023-05-31

**Authors:** Youngnim Shin, Deborah Cho, Seul Ki Kim, Jang-Soo Chun

**Affiliations:** National Creative Research Initiatives Center for Osteoarthritis Pathogenesis and School of Life Sciences, Gwangju Institute of Science and Technology, Gwangju, 61005 Republic of Korea

## Abstract

**Background:**

This study was performed to develop therapeutic targets of osteoarthritis (OA) that can be targeted to alleviate OA development (i.e., cartilage destruction) and relieve the OA-associated joint pain.

**Methods:**

The candidate molecule, STING (stimulator of interferon genes, encoded by Sting1), was identified by microarray analysis of OA-like mouse chondrocytes. Experimental OA in mice was induced by destabilization of the medial meniscus (DMM). STING functions in OA and hindpaw mechanical allodynia were evaluated by gain-of-function (intra-articular injection of a STING agonist) and loss-of-function (Sting1−/− mice) approaches.

**Results:**

DNA damage was observed in OA-like chondrocytes. Cytosolic DNA sensors, STING and its upstream molecule, cGAS (cyclic GMP-AMP synthase), were upregulated in OA chondrocytes and cartilage of mouse and human. Genetic ablation of STING in mice (Sting1−/−) alleviated OA manifestations (cartilage destruction and subchondral bone sclerosis) and hindpaw mechanical allodynia. In contrast, stimulation of STING signaling in joint tissues by intra-articular injection of cGAMP exacerbated OA manifestations and mechanical sensitization. Mechanistic studies on the regulation of hindpaw mechanical allodynia revealed that STING regulates the expression of peripheral sensitization molecules in the synovium and meniscus of mouse knee joints.

**Conclusion:**

Our results indicated that STING, which senses damaged cytosolic DNA and accordingly activates the innate immune response, regulates OA pathogenesis and hindpaw mechanical allodynia. Therefore, inhibition of STING could be a therapeutic approach to inhibit OA cartilage destruction and relieve the associated mechanical sensitization in model mice.

**Supplementary Information:**

The online version contains supplementary material available at 10.1186/s13075-023-03075-x.

## Background

Osteoarthritis (OA) is a whole-joint disease that involves cartilage destruction, osteophyte formation, synovial inflammation, subchondral bone sclerosis, etc. [1, 2], and arises from multiple etiologies, such as mechanical stress, metabolic stress, aging, and low-grade inflammation [3–5]. In addition to the above-listed OA manifestations, OA-associated joint pain is a direct cause of poor quality of life among patients and is the reason why they seek medical attention. The causes of OA-associated pain are not fully understood, but they are thought to include not only biochemical changes in the joint tissues but also alterations in the nervous system [1, 6]. The available options for treating OA pain remain relatively limited, due to inadequate efficacy and adverse effects from prolonged treatment. Therefore, it is becoming important to develop therapeutic targets for OA whose modulation can regulate both OA manifestations and mechanical sensitization.

In preliminary experiments, we performed bioinformatic analysis of various microarray datasets obtained from OA-like chondrocytes, seeking to identify possible therapeutic targets that regulate various OA manifestations and associated pain. We initially identified STING (stimulator of interferon genes, encoded by *Sting1*), also known as transmembrane protein 173 (TMEM173) and MPYS/MITA/ERIS, and IFI204 (interferon activated gene 204, a murine ortholog of human IFI16) as being specifically upregulated in chondrocytes exposed to interleukin (IL)-1β treatment or overexpressing cellular mediators of OA pathogenesis, such as hypoxia-inducible factor (HIF)-2α [7] or the zinc importer ZIP8 [8]. STING and IFI204 are pattern recognition receptors (PRRs) that sense cytosolic DNA as damage-associated molecular patterns (DAMPs) [9]. The sensing of cytosolic nucleic acids through binding with PRRs is known to promote inflammatory responses mediated by the innate immune response [10]. In addition to its critical function as an element of cytosolic DNA sensors, STING also regulates the innate immune response caused by cytosolic DNA [9, 11]. Cytosolic DNA is sensitized by cyclic GMP-AMP synthase (cGAS), which produces 2',3' cyclic GAMP (cGAMP), an agonist of STING [11]. STING activated by the binding of cGAMP stimulates TBK1 (TANK binding kinase 1); this causes activation of IRF3 or NF-κB to promote the innate immune response through the expression of type I interferons (IFNs) or cytokines, respectively [11].

A recent report found that lentivirus-mediated knockdown of STING in joint tissues alleviates DMM (destabilization of the medial meniscus)-induced cartilage destruction in mice [12]. A cell-based in vitro study suggested that STING regulates cellular senescence, apoptosis, and extracellular matrix degradation via activation of the NF-κB signaling pathway [12]. STING also acts as a regulator of nociception, and knockout of *Sting1* in mouse peripheral sensory neurons was reported to increase sensitivity to nociceptive stimuli and intrinsic excitability [13]. However, the role of STING in mechanical sensitization has not previously been evaluated. Here, we examined whether STING could be a therapeutic target capable of modulating various OA manifestations and OA-associated joint pain. We report that STING is a critical regulator of OA cartilage destruction and subchondral bone sclerosis as well as OA-associated mechanical allodynia in mice.

## Methods

### Human OA cartilage

Human OA cartilage (*n* = 5) was sourced from individuals undergoing arthroplasty [14, 15]. Characteristics of individuals with OA from whom cartilage samples were taken were presented in Supplementary Table 1. The Institutional Review Board of Wonkwang University Hospital approved the use of these materials, and all participants provided written informed consent before the operative procedure.

### Experimental OA in mice

Post-traumatic experimental OA was induced by DMM surgery on the right knee, and sham operation was performed on the left knee of the same mouse [16]. DMM surgery was performed in 12-week-old male C57BL/6 J mice (wild-type). We also used C57BL/6 J-background *Sting1*^*−/−*^ mice (B6(Cg)-*STING1*^*tm1.2Camb*^/J) purchased from the Jackson Laboratories (Bar Harbor, ME) (Supplementary Fig. 1). Mice were sacrificed at 6 or 8 weeks after DMM surgery and subjected to histological analyses based on the previous experiments [7, 8, 14, 15]. To stimulate STING signaling in joint tissues, 12-week-old male C57BL/6 J mice were IA injected once a week with cyclic GMP-AMP (cGAMP; 10 or 20 μg in 10 μl PBS) [17] for 3 weeks, and sacrificed at 3 or 8 weeks after the first IA injection. Alternatively, cGAMP was IA injected to sham- or DMM-operated mice at the time points indicated for each experiment, and each group of mice was randomly assigned and sacrificed at 6 weeks or 8 weeks respectively after DMM for histological analysis or von Frey test. PBS was IA injected as a control for cGAMP injection. The experimental design and numbers in each group are presented in Supplementary Fig. 2. All mice were maintained under specific pathogen-free conditions and all animal experiments were approved by the Gwangju Institute of Science and Technology Animal Care and Use Committee.

### von Frey test

Hindpaw mechanical allodynia was examined using the von Frey assay [14, 18, 19]. The behavior tests were performed at the indicated weeks after surgery. Mice were placed in an acrylic chamber above a metal grid floor and allowed to acclimatize to the test apparatus for at least 15 min. When the mouse ceased exploratory behavior, a von Frey filament was pressed against the plantar surface of the paw until the filament buckled, and held there for a maximum of 3 s. A positive response was noted if the paw was sharply withdrawn on application of the filament or there was flinching upon removal of the filament. Mice were randomly assigned to be tested by two blinded observers with a simplified up-down method [19].

### Histological analysis

Human OA cartilage and mouse joint tissues were fixed in 4% paraformaldehyde, decalcified in 0.5 M EDTA (pH 8.0), embedded in paraffin, sectioned at 5-μm thickness, and stained with safranin-O and hematoxylin [14, 15]. Images were acquired by an Aperio CS2 slide scanner (Leica Biosystems, Richmond, IL). OARSI grade and osteophyte maturity were calculated as the average scores obtained from three different sections selected at ~ 100-μm intervals. Each section was scored by four blinded observers, and the results are presented as the average value obtained. OARSI grade was expressed as the maximum score observed among the medial femoral condyle, medial tibial plateau, lateral femoral condyle, and lateral tibial plateau [20]. Osteophyte formation was identified by safranin-O staining, and osteophyte maturity (grade 0–3) was scored as described previously [21]. Subchondral bone thickness (SBP) was measured as the average length obtained from three different sections to assess subchondral bone sclerosis, using an Aperio Image Scope (Leica Biosystems) [22]. The results are presented as average values obtained from three joint sections.

### Immunohistochemistry and immunofluorescence microscopy

Immunostaining of STING and γH2AX (phosphorylated form of histone variant H2AX) in human and mouse joint sections was performed using a Dako LSAB2 horseradish peroxidase kit (Agilent, Santa Clara, CA). Briefly, slide sections were incubated overnight at 4℃ with rabbit anti-STING (Proteintech, Rosemont, IL) or rabbit anti-γH2AX (Cell Signaling Technology, Danvers, MA). The sections were incubated with Dako Envision^+^ System HRP-labeled polymer reagents, and immunoreactive proteins were visualized using the Dako AEC high-sensitivity substrate chromogen solution (Agilent). Images were acquired under an Aperio CS2 slide scanner. The IHC images of STING and γH2AX were semi-quantified by using the Positive Pixel Count version 9 of the Aperio ImageScope Viewer software. All three staining intensity ranges (weak, positive, and strong) were considered as positive staining. The percentage of positive pixels was calculated relative to the total number of pixels in the sections [23]. For immunofluorescence microscopy of TRPV1 (transient receptor potential vanilloid 1), CGRP (calcitonin gene-related peptide), and NGF (nerve growth factor) in mouse joint sections, the samples were incubated overnight at 4℃ with rabbit anti-TRPV1, goat anti-CGRP, or rabbit anti-NGF polyclonal antibodies (Abcam, Cambridge, MA). The samples were incubated with AlexaFluor 488-conjugated goat anti-rabbit IgG or AlexaFluor 555-conjugated donkey anti-goat IgG (Thermo Fisher Scientific, Waltham, MA). Detailed information for antibodies is presented in Supplementary Table 2. Fluorescence images were acquired by a VS200 research slide scanner (Olympus, Tokyo, Japan). Fluorescence intensity in immunostaining images of CGRP, TRPV1, and NGF was semi-quantified by using ImageJ program [24]. Briefly, eight region-of-interests (ROIs) containing multiple cells were randomly selected from 800X magnification of tissue sections. The integrated density was presented as the average value of eight ROIs. To obtain background fluorescence in one ROI, three non-cellular staining areas were selected and the integrated density was presented as the average value of three non-cellular staining areas. The corrected total cell fluorescence (CTCF) values were calculated using the formula: CTCF = integrated density in ROI – (area of selected ROI × mean intensity of background). The values for integrated density, area, and mean background density were obtained from the “measurement” function in ImageJ. We presented normalized CTCF in which CTCF values were divided by area of ROI (μm^2^).

### Primary culture of mouse articular chondrocytes

Chondrocytes were isolated from the femoral condyles and tibia plateaus of both knees from ~ 10 5-day-old ICR mouse pups [25]. Pooled chondrocytes were plated at 3 × 10^5^ cells per 35-mm dish in Dulbecco’s modified Eagle’s medium (DMEM; Gibco, Waltham, MA) supplemented with 10% fetal bovine serum and antibiotics. Cells were treated with IL-1β or infected with the indicated multiplicity of infection (MOI) of empty adenovirus (Ad-C) or adenovirus expressing hypoxia-inducible factor (HIF)-2α (Ad-HIF-2α) [7] or ZIP8 (Ad-ZIP8) [8], all of which were purchased from Vector Biolabs (Malvern, PA).

### Reverse transcription-polymerase chain reaction (RT-PCR) and quantitative RT-PCR (qRT-PCR) analysis

Total RNA was extracted from mouse chondrocytes using the TRI reagent (Molecular Research Center Inc., Cincinnati, OH) and reverse transcribed, and the resulting cDNA was amplified by PCR. The sequences of the PCR primers are presented in Supplementary Table 3. qRT-PCR was performed in a CFX Connect Real-Time PCR Detection System (Bio-Rad, Hercules, CA) using SYBR Premix Ex Taq (TaKaRa Bio Inc., Shiga, Japan). The 2^−ddCt^ method was used to analyze the relative gene expression levels.

### Western blotting

Total cell lysates were prepared using a lysis buffer containing cocktails of phosphatase inhibitors and proteinase inhibitors (Promega, Madison, WI). Proteins were fractionated by SDS-PAGE, transferred to a nitrocellulose membrane, and incubated with antibodies against γH2AX, cGAS (both from Cell Signaling Technology), STING (Proteintech), or ERK (BD Biosciences, San Jose, CA). The utilized secondary antibody was either goat anti-rabbit or horse anti-mouse IgG (Cell Signaling Technology) conjugated with HRP, and detection was performed using an ECL system (Thermo Fisher Scientific). Detailed information for antibodies is presented in Supplementary Table 2.

### Microarray analysis

Microarray data from primary-culture mouse chondrocytes treated with IL-1β or overexpressing HIF-2α (Ad-HIF-2α infection) or ZIP8 (Ad-ZIP8 infection) were deposited to the Gene Expression Omnibus under accession codes GSE104794 (HIF-2α), GSE104795 (ZIP8), and GSE104793 (IL-1β). Detailed methods for the generation of microarray data were previously reported [14]. We screened these microarray data to evaluate differential expression patterns of cytosolic DNA or RNA sensors that could be associated with OA pathogenesis.

### Statistical analysis

For statistical comparison of experimental groups, the data were analyzed by the Shapiro–Wilk test for normality and Levene’s test for homogeneity of variance. Parametric data were compared by paired t-test for qRT-PCR and semi-quantification of IHC images, Student’s t-test for hindpaw withdrawal threshold and IF quantification, and one-way analysis of variance (ANOVA) with Bonferroni’s *post-hoc* test for SBP thickness when data followed a normal distribution. Non-parametric data based on the ordinal grading systems (OARSI grade and osteophyte maturity) were compared using the Mann–Whitney U test for two groups and Kruskal–Wallis with Bonferroni’s *post-hoc* test for more than two groups were used when normality was not obtained. Values for parametric data are shown as mean ± 95% CI (Confidence Interval) or ± s.e.m with* P*-value, whereas values for non-parametric data are shown as median ± interquartile range (IQR) with *P*-value. Significance was accepted at the 0.05 level of probability (*p* < 0.05).

## Results

### The cytosolic DNA sensors, IFI204 and STING, are upregulated in OA-like mouse chondrocytes.

**Fig. 1:**
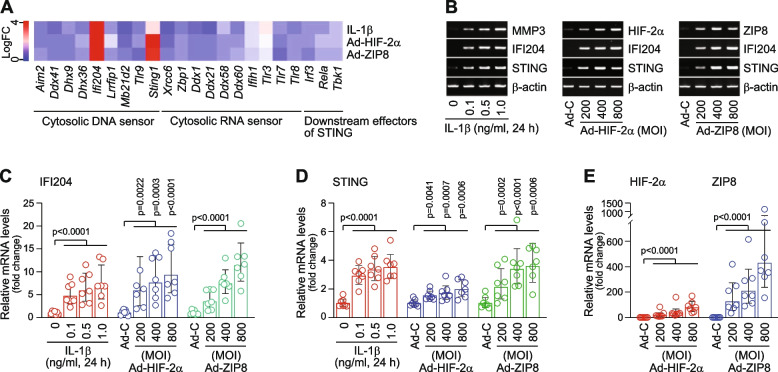
Upregulation of the cytosolic DNA sensors, IFI204 and STING, in OA-like mouse chondrocytes. **A** Heat map of cytosolic nucleic acid sensors in chondrocytes treated with IL‐1β (1 ng/ml, 36 h) or infected with 800 MOI of Ad-HIF‐2α or Ad-ZIP8 (36 h). **B**-**E** Representative RT-PCR images (**B**) and relative mRNA levels (**C**–**E**) of the indicated molecules in mouse chondrocytes treated with IL-1β or infected with the indicated MOI of Ad-C (control), Ad-HIF-2α, or Ad-ZIP8 for 36 h. Values are presented as mean ± 95% CI, and significance was evaluated by paired t-test (*n* = 7)

We first screened the mRNA levels of cytosolic DNA sensors, cytosolic RNA sensors, and related molecules from microarray data obtained from OA-like chondrocytes. OA chondrocytes were mimicked by treating primary-culture mouse chondrocytes with the pro-inflammatory cytokine, IL-1β [26], or an adenovirus expressing a critical cellular mediator of OA pathogenesis, such as HIF-2α (Ad-HIF-2α) [7] or ZIP8 (Ad-ZIP8) [8]. Our microarray analysis revealed that the mRNA levels of interferon-inducible gene IFI204 (the murine ortholog of human IFI16) and stimulator of interferon genes (STING) were markedly increased in OA-like chondrocytes (Fig. 1A). (q)RT-PCR analysis further confirmed that IL-1β treatment or overexpression of HIF-2α or ZIP8 in chondrocytes significantly increased the mRNA levels of IFI204 and STING (Fig. 1B-E). STING is not only a cytosolic DNA sensor, but also an essential mediator of innate immune responses initiated by cytoplasmic DNA [9, 11, 27]. We, therefore, focused on the possible functions of upregulated STING in chondrocytes during OA pathogenesis and OA-associated pain behavior in mouse.

**Fig. 2:**
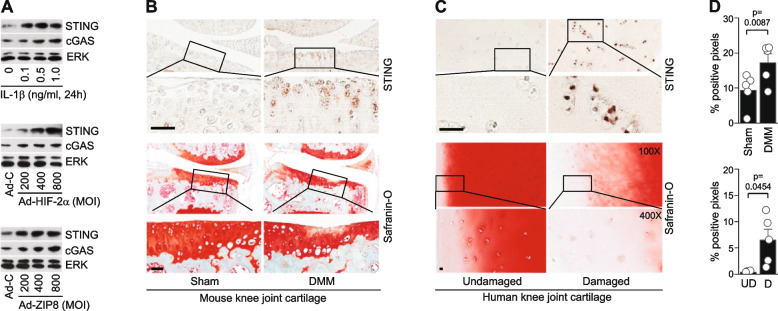
Upregulation of STING in mouse and human OA chondrocytes. **A** Representative Western blot images of STING and cGAS in chondrocytes treated with IL‐1β (1 ng/ml, 36 h) or infected with 800 MOI of Ad-HIF‐2α or Ad-ZIP8 (36 h). ERK was detected as a loading control. **B** and **C** Representative images of safranin-O staining and STING immunostaining in DMM-operated mouse OA cartilage (*n* = 5 mice) (**B**) and human OA cartilage tissue (*n* = 5 patients) (**C**). **D** Semi-quantification of STING positive pixels determined by the Aperio ImageScope Viewer software (*n* = 5 mice or patients). Values are presented as mean ± s.e.m., and significance was evaluated by paired t-test. Scale bars: 50 µm

Similar to the results obtained when we examined the mRNA levels, IL-1β treatment or overexpression of HIF-2α or ZIP8 increased the protein levels of STING in primary-culture mouse chondrocytes (Fig. 2A). The protein levels of cGAS, which is an upstream molecule of STING [11], were also increased in these chondrocytes (Fig. 2A). Consistently, STING protein levels were markedly elevated in post-traumatic OA cartilage caused by DMM surgery in mouse (Fig. 2B and D). Similarly, compared to an undamaged region of the same cartilage tissue, a damaged part of human OA cartilage exhibited markedly elevated STING protein levels (Fig. 2C and D). These results suggest that activation of innate immune response by the cGAS and STING in chondrocytes plays a role in OA pathogenesis.

**Fig. 3:**
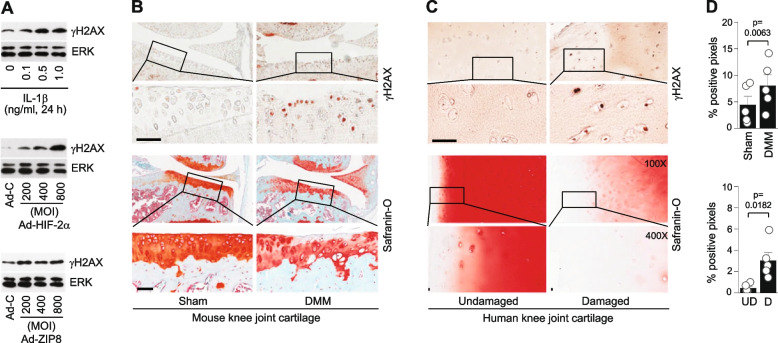
DNA damage in mouse and human OA chondrocytes. **A** Representative Western blot images of γH2AX in chondrocytes treated with IL‐1β (1 ng/ml, 36 h) or infected with 800 MOI of Ad-HIF‐2α or Ad-ZIP8 (36 h). ERK was detected as a loading control. **B** and **C** Representative images of safranin-O staining and γH2AX immunostaining in mouse OA cartilage induced by DMM surgery (*n* = 5) (**B**) and human OA cartilage tissue (*n* = 5 patients) (**C**). **D** Semi-quantification of γH2AX positive pixels determined by the Aperio ImageScope Viewer software (*n* = 5 mice or patients). Values are presented as mean ± s.e.m., and significance was evaluated by paired t-test. Scale bars: 50 µm

The cGAS-STING pathway activates an innate immune response by sensing cytosolic DNA as a DAMP signal [10, 11]. Endogenous cytosolic DNA can come from damaged mitochondrial DNA or nuclear DNA that is leaked/damaged by chromosome instability and cell damage [11, 26]. We, therefore, evaluated DNA damage in OA-like chondrocytes and OA cartilage by detecting γH2AX, a marker for DNA damage [28]. The protein level of γH2AX was markedly increased in primary-culture mouse chondrocytes stimulated with IL-1β or overexpressing HIF-2α or ZIP8 (Fig. 3A). γH2AX was also found to be upregulated in DMM-operated post-traumatic OA cartilage of mouse (Fig. 3B and D) and damaged parts of human OA cartilage compared to the corresponding undamaged regions of the same cartilage tissues (Fig. 3C and D). These results suggest that catabolic factors such as IL-1β, HIF-2α, or ZIP8 induce DNA damage in OA chondrocytes, and activates cGAS and STING during OA pathogenesis.

### Genetic ablation of STING in mouse (*Sting1*^*−/−*^) mitigates post-traumatic OA and mechanical allodynia

**Fig. 4:**
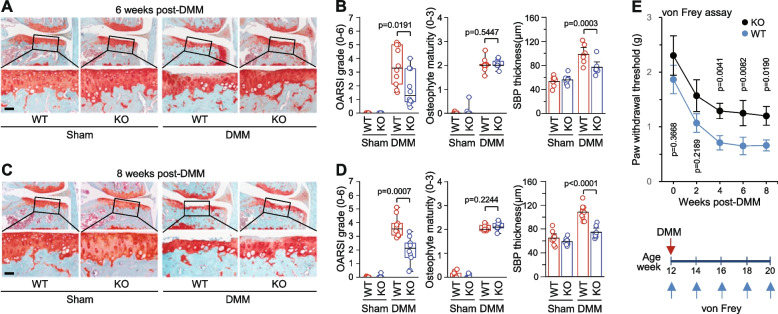
Genetic ablation of *Sting1* mitigates OA cartilage destruction and mechanical allodynia in mouse. **A** and **B** Representative safranin-O staining images (**A**) and OARSI grade, osteophyte maturity, and SBP thickness (**B**) in sham- or DMM-operated WT and *Sting1*^*−/−*^ mice at 6 weeks after DMM surgery (*n* = 10 mice per group). **C** and **D** Representative safranin-O staining images (**A**) and OARSI grade, osteophyte maturity, and SBP thickness (**B**) in sham- or DMM-operated WT and *Sting1*^*−/−*^ mice at 8 weeks after DMM surgery (*n* = 10 mice per group). **E** von Frey assay in WT and *Sting1*^*−/−*^ mice at the indicated weeks post-DMM surgery (*n* = 15 mice per group). Data for OARSI grade and osteophyte maturity are presented as median ± interquartile range (IQR) and paw withdrawal threshold is presented as mean with s.e.m., and significance was evaluated by Mann–Whitney U test and Student *t*-test, respectively. Values for SBP thickness are presented as mean ± 95% CI, and significance was assessed by one-way ANOVA with *post-hoc* Bonferroni test. Scale bars: 50 µm

The role of STING in OA pathogenesis was directly examined by DMM surgery in WT and *Sting1*^*−/−*^ mice. *Sting1*^*−/−*^ mice are viable and exhibited normal development of skeletal elements (Supplementary Fig. 1). Compared with WT mice, *Sting1*^*−/−*^ mice exhibited significant reduction in cartilage destruction at both 6 and 8 weeks after DMM surgery. For instance, at 6 weeks after DMM surgery, median OARSI grade in WT mice [3.29 (IQR 2.11–5.00)] was decreased to 1.28 (IQR 0.83—3.25, *P* = 0.0191) in *Sting1*^*−/−*^ mice (Fig. 4A − D). Similarly, at 8 weeks after DMM surgery, 3.53 (IQR 3.08—3.94) median OARSI grade in WT mice was decreased to 2.11 (IQR 1.44—2.50, *P* = 0.0007) in *Sting1*^*−/−*^ mice (Fig. 4A − D). Thickening of the subchondral bone plate (SBP), an indicator of subchondral bone sclerosis [8, 14, 22], was also inhibited in DMM-operated *Sting1*^*−/−*^ mice. SBP thickness in WT mice were 99.47 ± 5.03 μm (95% CI [76.03–121.81]) and 108.26 ± 3.85 μm (95% CI [92.51–132.24]) at 6 and 8 weeks after DMM surgery. These values were significantly decreased to 74.90 ± 3.69 μm (95% CI [59.19–103.05], *P* = 0.0003) and 74.62 ± 2.94 μm (95% CI [63.87–91.19], *P* < 0.0001) in *Sting1*^*−/−*^ mice at 6 or 8 weeks, respectively (Fig. 4A − D). However, DMM-induced osteophyte formation was not modulated by genetic ablation of STING at either time point (Fig. 4A − D).

Although the function of STING in OA-associated mechanical allodynia was previously unknown, STING was recently shown to regulate nociception via type-I interferon (IFN-I) signaling in peripheral sensory neurons [13]. We, therefore, examined whether STING regulates mechanical sensitivity in WT and *Sting1*^*−/−*^ mice (KO) during OA progression. Our von Frey assay in DMM-operated mice revealed that *Sting1*^*−/−*^ mice were less sensitive to von Frey filaments before the initiation of the surgery and showed significant increase in mechanical threshold from 4 to 8 weeks after DMM surgery (Fig. 4E). Mechanical sensitivity was not significantly different in sham-operated WT and KO mice (Supplementary Fig. 3). These findings indicate that STING deficiency plays protective roles in DMM-induced post-traumatic OA and mechanical allodynia in mice.

### Stimulation of the STING pathway exacerbates OA pathogenesis and the associated mechanical allodynia

**Fig. 5:**
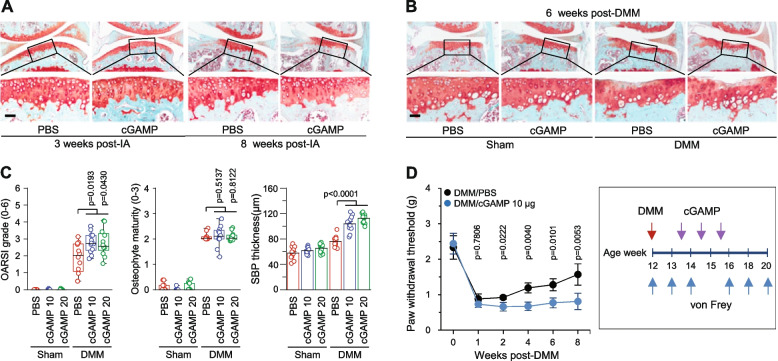
Stimulation of the STING in mouse knee joints exacerbates post-traumatic OA cartilage destruction and OA-associated mechanical sensitivity. **A** Wild-type (WT) mice were IA injected with 20 μg of cGAMP or PBS once weekly for 3 weeks, and sacrificed at 3 weeks or 8 weeks after the first IA injection. Presented are representative safranin-O staining images of joint sections (**A**, *n* = 5 mice/10 knees per group). **B** and **C** Sham- or DMM-operated WT mice were IA injected once a week for 3 weeks with the indicated concentrations of cGAMP (μg) or PBS starting at 10 days after DMM surgery. Presented are representative safranin-O staining images (**B**) and OARSI grade, osteophyte maturity, and SBP thickness (**C**). **D** von Frey assay at the indicated weeks post-DMM surgery in mice IA injected with cGAMP or vehicle (PBS) (*n* = 15 mice per group). Values in C and D are presented as median ± interquartile range (IQR) for OARSI grade and osteophyte maturity, mean ± s.e.m for paw threshold, and mean ± 95% CI for SBP thickness. Significance was evaluated by Kruskal–Wallis with *post-hoc* Bonferroni test for OARSI grade and osteophyte maturity, Student *t*-test for paw threshold, and one-way ANOVA with *post-hoc* Bonferroni test for SBP thickness. Scale bars: 50 µm

Next, we investigated whether stimulation of the STING in joint tissues modulates OA pathogenesis and mechanical sensitivity. For this purpose, cGAMP, a natural agonist of STING [17], was IA injected into the knee joints of mice with or without DMM surgery. IA injection of cGAMP alone did not cause any OA-like change in the joint tissues at 3 and 8 weeks post-IA injection (Fig. 5A; Supplementary Fig. 2A). However, IA injection of cGAMP in DMM-operated knee joints significantly exacerbated cartilage destruction. Compared to vehicle-treated group, IA injection of 10 or 20 μg of cGAMP increased median OARSI grade from 2.03 (IQR 1.09 – 2.71) to 2.73 (IQR 2.32 – 3.21, *P* = 0.0193) and 2.57 (IQR 2.32 – 3.32, *P* = 0.0430), respectively (Fig. 5B and C; Supplementary Fig. 2B). IA injection of cGAMP in DMM-operated mice also caused more significant SBP thickening. For instance, SPB thickness in PBS-injected mice was 75.95 ± 2.33 μm (95% CI [64.83–94.85]), whereas these value was increased to 103.93 ± 3.49 μm (95% CI [83.00–122.52], *P* < 0.0001) and 112.35 ± 2.03 μm (95% CI [99.37–122.36], *P* < 0.0001) by IA injection of 10 and 20 μg of cGAMP, respectively (Fig. 5B and C). Contrast to OARSI grade and SBP thickening, osteophyte formation was not modulated by IA injection of cGAMP in DMM-operated mice (Fig. 5B and C). Together with the data obtained from *Sting1*^*−/−*^ mice, our results indicate that STING regulates OA cartilage destruction and subchondral bone sclerosis, but not osteophyte formation, in DMM-operated mice.

Consistent with the observation of increased mechanical threshold in DMM-operated *Sting1*^*−/−*^ mice, stimulation of STING via IA injection of cGAMP in DMM-operated knee joints significantly exacerbated OA-associated mechanical sensitivity in mice (Fig. 5D). In contrast, IA injection of cGAMP in sham-operated knee joints did not cause significant differences in mechanical sensitivity (Supplementary Fig. 4). These results are consistent with the view that inhibition of STING yields a chondro-protective function and reduces mechanical allodynia in DMM-operated mice (Fig. 4A − E).

### STING regulates the expression of pain-sensitization molecules

**Fig. 6:**
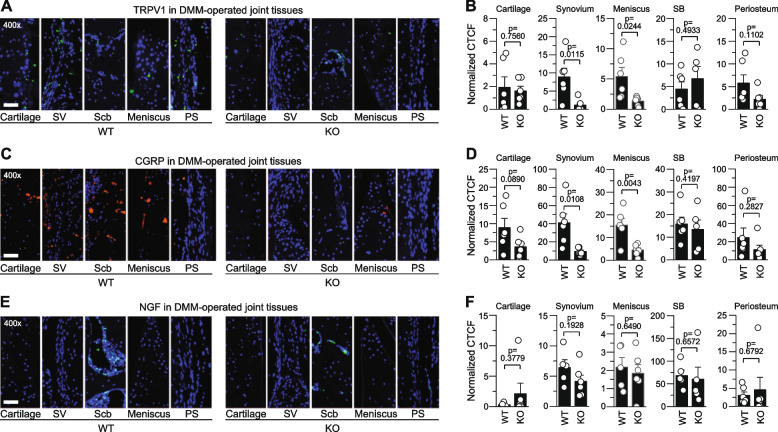
Decreased expression of pain-sensitizing molecules in joint tissues of DMM-operated *Sting1*^*−/−*^ mice. **A** and **B** Representative immunostaining images (**A**) and quantification (**B**) of TRPV1 in the indicated tissues of DMM-operated WT and *Sting1*^*−/−*^ mice (*n* = 6 mice per group). **C** and **D** Representative immunostaining images (**C**) and quantification of CGRP (**D**) in DMM-operated WT and *Sting1*^*−/−*^ mice (*n* = 6 mice per group). **E** and **F** Representative immunostaining images (**E**) and quantification (**F**) of NGF in DMM-operated WT and *Sting1*^*−/−*^ mice (*n* = 6 mice per group). The corrected total cell fluorescence (CTCF) is calculated by subtracting the contribution of background intensity from the integrated fluorescence density within the ROIs (**B**, **D**, **F**). CTCF values were normalized by area of ROI, presented as mean ± s.e.m., and significance was assessed by Student’s t-test. SV: synovium, Scb: subchondral bone, PS: periosteum. Scale bars: 50 µm

In an attempt to elucidate the regulatory mechanisms underlying the impact of STING on alleviation of mechanical allodynia, we examined whether STING regulates the expression of molecules involved in pain sensitization, such as TRPV1 [29, 30], CGRP [31, 32], and NGF [33, 34]. These molecules play a key role in peripheral sensitization by activating and sensitizing nociceptors, which contribute to the development of OA-associated pain [35, 36]. We compared the expression levels of these molecules in the cartilage, meniscus, synovium, subchondral bone, and periosteum of DMM-operated *Sting1*^*−/−*^ mice and WT. Immunofluorescence microscopy of knee joint sections revealed that the protein levels of TRPV1 were significantly reduced in the synovium and meniscus of *Sting1*^*−/−*^ mouse knee joints (Fig. 6A and B; Supplementary Fig. 5A). CGRP protein levels were also significantly decreased in the synovium and meniscus of *Sting1*^*−/−*^ mice (Fig. 6C and D; Supplementary Fig. 5B). In contrast, the expression levels of these molecules were not modulated by STING deficiency in other examined tissues, such as cartilage, subchondral bone, and periosteum (Fig. 6A ~ D; Supplementary Fig. 5A and B; Supplementary Fig. 6A and B). Unlike the findings for TRPV1 and CGRP, the expression levels of NGF in the examined tissues were not modulated in DMM-operated *Sting1*^*−/−*^ mice compared to WT (Fig. 6E and F; Supplementary Figs. 5C and 6C). Finally, expression of TRPV1, CGRP, and NGF was not modulated in sham-operated WT and *Sting1*^*−/−*^ mice (Supplementary Fig. 7). These results suggest a possibility that STING may regulate OA-associated mechanical allodynia by decreasing the expression of peripheral sensitization molecules in the synovium and meniscus of mouse knee joints.

## Discussion

OA is recognized as a chronic and low-grade inflammatory disease associated with innate immune responses [2, 4, 5]. The pathology is primarily regulated by damage-associated molecular patterns (DAMPs) and pattern recognition receptors (PRRs) [10]. Here, we demonstrated that cGAS and STING in chondrocytes, which recognize damaged DNA and thereby activates an innate immune response, is a critical regulator of OA pathogenesis and the associated pain behavior in mice. We first demonstrated that OA chondrocytes exhibit DNA damage and upregulation of cGAS and STING proteins. Cytosolic DNA, which can be derived from DNA damage, is classified as a DAMP and can be sensed by cGAS. The production of cGAMP by cGAS activates STING and its downstream signaling events, such as the production of type 1 interferons (IFNs) and many other inflammatory cytokines [11, 27]. Therefore, our results support the notion that upregulation and activation of the cGAS and STING in OA chondrocytes, possibly by cytosolic nucleic acids derived from DNA damage, regulates OA cartilage destruction, subchondral bone sclerosis, and OA-associated joint pain in mice. In addition to STING, we found that the cytosolic DNA sensor, IFI204, is also upregulated in OA chondrocytes. However, while adenoviral overexpression of STING in primary-culture chondrocytes upregulated matrix-degrading enzymes (MMP3 and MMP13), a cytokine (IL-6), and chemokines (CCL2, CCR2, CXCL10, etc.) and downregulated extracellular matrix molecules (type II collagen and aggrecan), we found that adenoviral overexpression of IFI204 did not modulate the expression levels of these molecules (data not shown). Furthermore, adenoviral overexpression of IFI204 in mouse joint tissues via IA injection did not cause any OA-like change, such as cartilage loss or synovitis (data not shown). Based on these preliminary observations, we focused on the role of STING in OA pathogenesis and related pain behavior.

Our results related to the role of STING in OA cartilage degradation resemble those recently reported by Guo et al. [12]. These authors also observed upregulation of STING in OA chondrocytes of mouse and human and found that knockdown of STING by IA injection of lentiviral particles encoding sh-STING alleviated DMM-induced post-traumatic OA cartilage destruction [12]. In the present study, we further validated the in vivo function of STING in OA pathogenesis by employing *Sting1*^*−/−*^ mice, and demonstrated that genetic ablation of STING significantly mitigates post-traumatic OA cartilage destruction. We also demonstrated that stimulation of STING by IA injection of cGAMP exacerbates DMM-induced cartilage destruction. Guo et al. [12] additionally used primary-culture mouse chondrocytes to show that the catabolic function of STING in OA cartilage destruction may be due to its capacity in chondrocytes to cause cellular senescence, apoptosis, upregulation of matrix-degrading enzymes, and downregulation of extracellular matrix molecules via activation of the NF-κB signaling pathway. Similarly, we observed that adenoviral overexpression of STING in chondrocytes upregulates matrix-degrading enzymes (MMP3 and MMP13), a cytokine (IL-6), and chemokines (CCL2, CCR2, CXCL10, etc.) and downregulates extracellular matrix molecules (type II collagen and aggrecan). These catabolic effects of STING might contribute to the observed OA cartilage destruction. OA is a whole-joint disease involving not only cartilage destruction but also other manifestations, such as osteophyte formation and subchondral bone sclerosis [1, 2]. Interestingly, we found that knockout or activation of STING in mice regulates cartilage destruction and subchondral bone sclerosis, as determined by thickening of the subchondral bone plate [22], but had no apparent effect on osteophyte formation. The mechanism underlying these differential effects of STING remains to be elucidated. Nevertheless, natural products (e.g., gelsevirine [37]) or metabolites (e.g., itaconate [38]) that inhibit the STING pathway have been found to exhibit therapeutic potential against OA cartilage destruction [37, 38].

A possible role of STING in OA-associated mechanical allodynia has not previously been reported. However, recent studies indicated that STING acts as a regulator of nociception, and knockout of STING in mouse peripheral sensory neurons reportedly increased the sensitivity to nociceptive stimuli and intrinsic excitability [13]. We demonstrate here that STING regulates OA-associated mechanical sensitivity in mice. However, in contrast to the effects observed for sensory neurons [13], we found that *Sting1*^*−/−*^ mice exhibit increased mechanical threshold and stimulation of STING in joint tissues exacerbated OA-associated mechanical sensitivity in mice. This difference may reflect the disease models utilized: We herein used post-traumatic OA induced by DMM, whereas the previous authors used a syngeneic bone cancer model induced by injecting a lung carcinoma cell line into bone. We also locally injected cGAMP into the articular cavity and measured hindpaw mechanical allodynia for long periods (up to 8 weeks), whereas Donnelly et al. [13] used intrathecal injection for systemic administration of cGAMP to mice and observed behavior daily for 4 days.

OA pain is classically considered to be a peripheral nociceptive sensitization pain [6, 35, 36]. The pain is associated with structural changes in joint tissues (e.g., bone marrow lesions, synovitis, and subchondral bone remodeling) and new nerve growth and innervation in the cartilage and meniscus of the damaged joint [6, 39]. Clinical reports also suggest that OA pain is driven by neuronal hyperexcitability induced by peripheral sensitization of the affected joint. For instance, IA injection of anesthetics can mitigate OA pain [40], and peripheral application of a neutralizing antibody against NGF shows analgesic effects in OA [41]. Total knee joint replacement, which eliminates peripheral nociceptive inputs, yields pain relief in the majority of cases [42]. Collectively, these results indicate that deletion of peripheral pain stimuli in local tissue could relieve OA pain. Therefore, modulation of the expression of peripheral sensitization molecules, such as TRPV1 and CGRP, in the synovium and meniscus of a mouse joint may contribute to the observed modulation of OA-associated mechanical allodynia in mice.

Nociceptors detect pain signals in the periphery and carry it to the dorsal horn of the spinal cord [6]. OA pain can originate from various joint tissues, as nociceptors are abundant in the synovium, ligaments, periosteum, meniscus, and subchondral bone [6]. Exposure of nociceptors to biochemical stimuli in joint tissues during OA progression contributes to the development of disease-associated pain [35, 36]. Because TRPV1 and CGRP play a key role in the peripheral sensitization by activating and sensitizing nociceptors [29, 31, 32], OA pain relief in *Sting1*^*−/−*^ mice appears to reflect the down-regulation of these molecules in the synovium and meniscus. Indeed, the expression of TRPV1 and CGRP are increased in the synovium of an OA joint, and this is correlated with increased OA pain [29, 31]. TRPV1 is a nonspecific cation channel that is well known for its function in nociception. Genetic ablation of TRPV1 in mice reduced pain in an adjuvant-induced arthritis model [30] and IP injection of the TRPV1 antagonists, A-889425 and JNJ-17203212, reduced pain behaviors in the monoiodoacetate-induced OA model [29, 43]. CGRP is a vasodilatory neuropeptide that is increased in the synoviocytes of human OA patients [44]. The degree of OA pain is correlated with the levels of CGRP in serum and synovial fluid in knee OA [9]. Additionally, CGRP-positive nerve fibers are observed in the menisci of patients with knee OA, and CGRP expression is increased in the synovial tissues of OA patients [32, 33, 45]. Together, these previous findings are consistent with our current observations that *Sting1*^*−/−*^ mice exhibit down-regulation of TRPV1 and CGRP in the synovium and meniscus and alleviation of mechanical sensitivity.

## Conclusions

In conclusion, we demonstrate in this study that upregulation and activation of STING is a critical regulator of OA pathogenesis and mechanical sensitivity in mice. Therefore, STING could be an effective therapeutic target to inhibit OA development and mechanical allodynia in a mouse model.

## Supplementary Information

**Additional file 1: Supplementary Table 1.** Characteristics of individuals with OA from whom cartilage samples were taken. **Supplementary Table 2.** List of primary and secondary antibodies. **Supplementary Table 3.** PCR primers and conditions. **Supplementary Fig. 1.** Characterization of *Sting1*^-/-^ mice. **Supplementary Fig. 2.** Experimental design and number of mice assigned to each group. **Supplementary Fig. 3.** Genetic ablation of *Sting1* mitigates mechanical sensitivity in mouse. **Supplementary Fig. 4.** Stimulation of the STING in mouse knee joints exacerbates OA-associated mechanical allodynia. **Supplementary Fig. 5.** Expression of pain-sensitizing molecules in joint tissues of DMM-operated WT and *Sting1*^-/-^ mice. **Supplementary Fig. 6.** Expression of pain-sensitizing molecules in periosteum of DMM-operated WT and *Sting1*^-/-^ mice. **Supplementary Fig. 7.** Expression of pain-sensitizing molecules in joint tissue of sham-operated WT and *Sting1*^-/-^ (KO) mice.

## Data Availability

The data supporting the conclusions of this article are included within the article.

## Abbreviations

cGAMP: Cyclic GAMP
cGAS: Cyclic GMP-AMP synthase
CGRP: Calcitonin gene-related peptide
γH2AX: Phosphorylated form of histone variant H2AX
HIF: Hypoxia-inducible factor
IL: Interleukin
KO: Knock-out
NGF: Nerve growth factor
STING: Stimulator of interferon genes
TRPV1: Transient receptor potential vanilloid 1
WT: Wild-type
OA: Osteoarthritis
