# Supplementary material for: STING mediates experimental osteoarthritis and mechanical allodynia in mouse
Source: Arthritis Res Ther. 2023 May 31;25:90. doi: 10.1186/s13075-023-03075-x (PMC10230703; doi:10.1186/s13075-023-03075-x)
Supplement: Supplementary file 1 — Additional file 1: Supplementary Table 1. Characteristics of individuals with OA from whom cartilage samples were taken. Supplementary Table 2. List of primary and secondary antibodies. Supplementary Table 3. PCR primers and conditions. Supplementary Fig. 1. Characterization of Sting1-/- mice. Supplementary Fig. 2. Experimental design and number of mice assigned to each group. Supplementary Fig. 3. Genetic ablation of Sting1 mitigates mechanical sensitivity in mouse. Supplementary Fig. 4. Stimulation of the STING in mouse knee joints exacerbates OA-associated mechanical allodynia. Supplementary Fig. 5. Expression of pain-sensitizing molecules in joint tissues of DMM-operated WT and Sting1-/- mice. Supplementary Fig. 6. Expression of pain-sensitizing molecules in periosteum of DMM-operated WT and Sting1-/- mice. Supplementary Fig. 7. Expression of pain-sensitizing molecules in joint tissue of sham-operated WT and Sting1-/- (KO) mice. [file 13075_2023_3075_MOESM1_ESM.pdf]

Supplementary Information

STING mediates experimental osteoarthritis and mechanical allodynia in mouse

Youngnim Shin, Deborah Cho, Seul Ki Kim, and Jang-Soo Chun

Supplementary Table 1. Characteristics of individuals with OA from whom cartilage samples were taken.

		Case 1	Case 2	Case 3	Case 4	Case 5
Age		82	64	65	72	63
Gender		Male	Male	Male	Female	Female
ICRS grade		4	4	4	4	4
Joint		Knee	Knee	Knee	Knee	Knee
Height (cm)		159	168	178	158	158
Body weight (kg)		56	84	90	71	71
Other disease[§]	RA	-	-	-	-	-
	DB	-	-	+	-	-
	HT	-	-	-	-	-
	Cancer	-	-	-	-	-

[§]RA: rheumatoid arthritis, DB: diabetes, HT: hypertension

Supplementary Table 2. List of primary and secondary antibodies

Antibody	Dilution	Cat. No.	Company
STING	1:100 (IHC)	19851-1-AP	Proteintech
STING	1:1000 (WB)	19851-1-AP	Proteintech
γH2AX	1:100 (IHC)	97185	Cell Signaling Technology
γH2AX	1:1000 (WB)	97185	Cell Signaling Technology
cGAS	1:1000 (WB)	31659	Cell Signaling Technology
ERK	1:4000 (WB)	91015	BD Bioscience
CGRP	1:500 (IF)	ab36001	Abcam
TRPV1	1:500 (IF)	ab31895	Abcam
NGF	1:500 (IF)	ab52918	Abcam
AlexaFluor 488	1:500 (IF)	A-11034	Thermofisher
AlexaFluor 555	1:500 (IF)	A-21432	Thermofisher

Supplementary Table 3. PCR primers and conditions.

mRNA	Species	Strand	Sequence (5´-3´)	At (℃)	Size (bp)
STING	Mouse	Sense<br>Antisense	5´-TCCTCCAAAACACTGCTGACA-3´<br>5´-GGGGCAGCATATCTCGGAAT-3´	60	339
IFI204	Mouse	Sense<br>Antisense	5´-GCATCTGAAAGAGGCGAGAC-3´<br>5´-GGGCTCTGAGTGGAGAACAG-3´	60	339
HIF-2α	Mouse	Sense<br>Antisense	5´-CGAGAAGAACGACGTGGTGTTC-3´<br>5´-GTGAAGGCTGGCAGGCTCC-3´	64	333
ZIP8	Mouse	Sense<br>Antisense	5´-GAACAATTGCCTGGATGATCACGC-3´<br>5´-AAGCCGGTTAACATCCCTGCATTC-3´	58	430
MMP3	Mouse	Sense<br>Antisense	5´-AGGGATGATGATGCTGGTATGG-3´<br>5´-CCATGTTCTCCAACTGCAAAGG-3´	58	434
β-actin	Mouse	Sense<br>Antisense	5´-ATATCGCTGCGCTGGTCGTC-3´<br>5´-AGGATGGCGTGAGGGAGAGC-3´	58	517

S, sense; As, antisense; At, annealing temperature

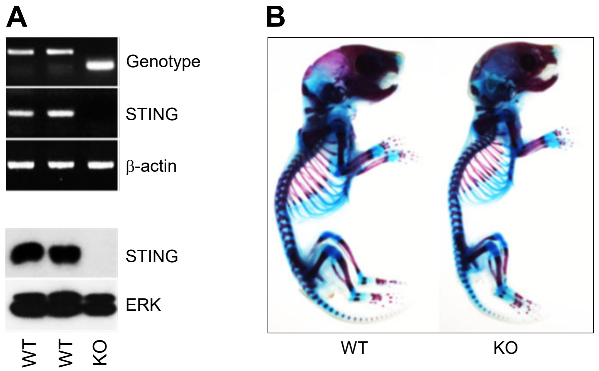

Supplementary Fig. 1. Characterization of *Sting1*$^{-/-}$ mice. (A) Genotype and mRNA and protein levels of STING in primary culture chondrocytes obtained from WT and *Sting1*$^{-/-}$ mice. (B) Representative skeletal staining images of E18.5 WT and *Sting1*$^{-/-}$ mouse embryos.

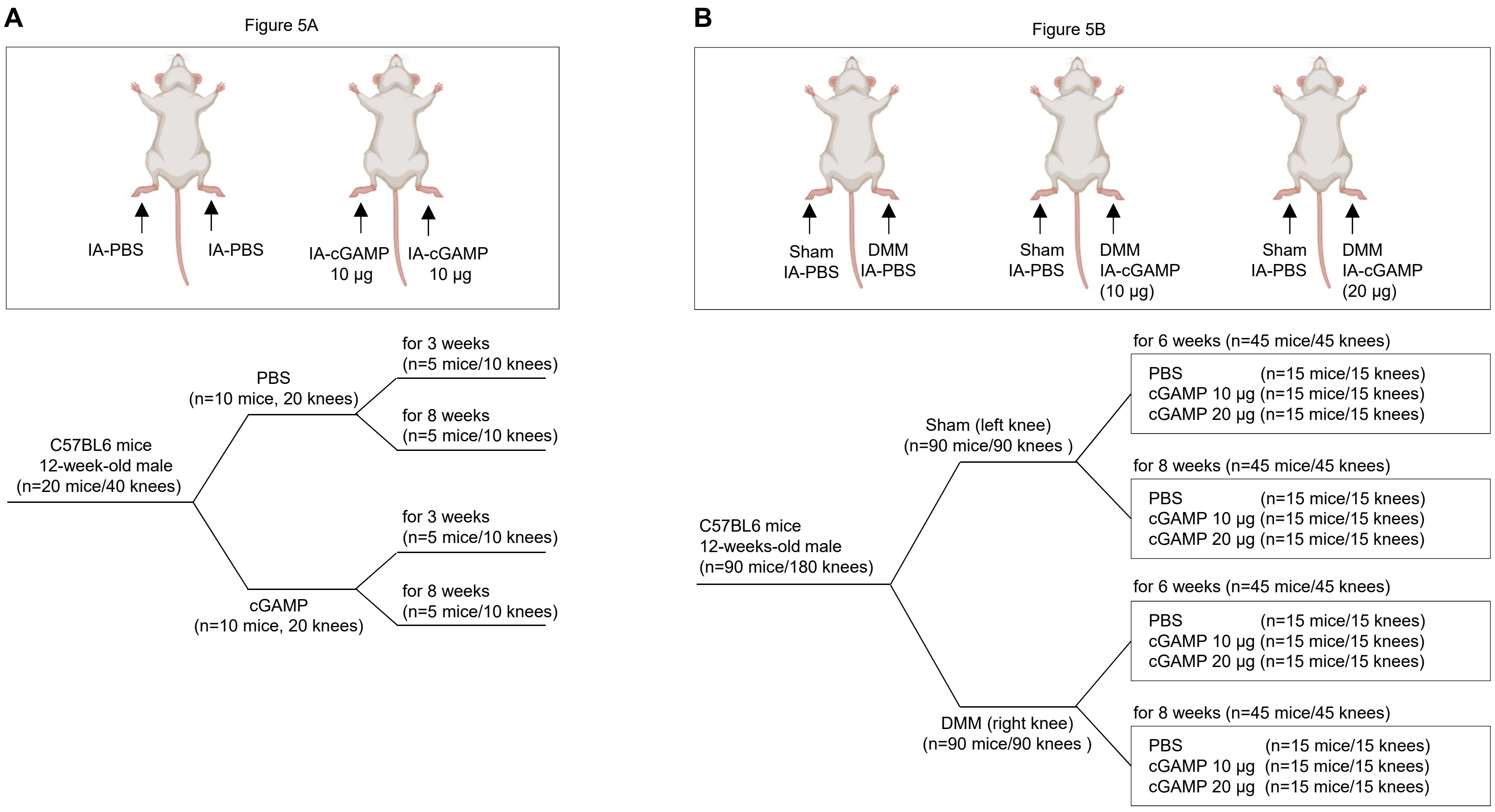

Supplementary Fig. 2. Experimental design and number of mice assigned to each group. (A) IA injection of cGAMP in mice. PBS or GAMP were IA injected in both knees of mice. (B) DMM surgery was performed on the right knee and sham operation was performed on the left knee of the same mouse. The number in A indicated the numbers of legs, whereas the numbers in B indicate the number of mice per group.

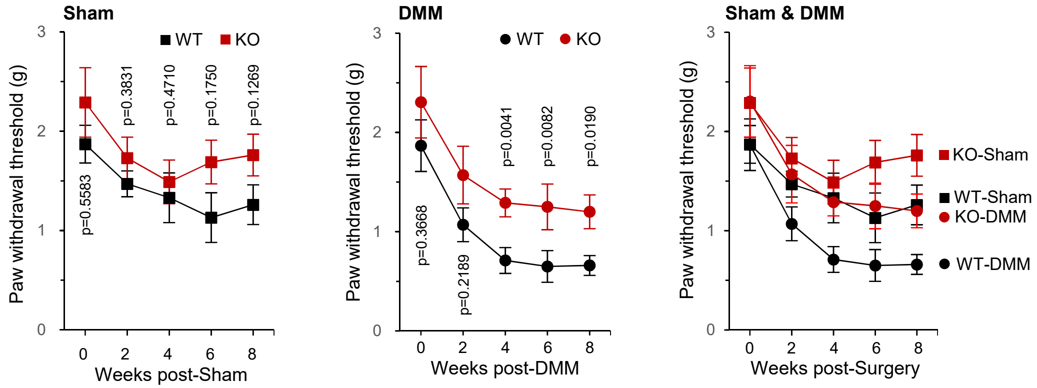

Supplementary Fig. 3. Genetic ablation of *Sting1* mitigates mechanical sensitivity in mouse. von Frey assays were performed in wild-type (WT) and *Sting1*$^{-/-}$ (KO) mice at the indicated weeks after sham operation or DMM surgery (n = 15 mice per group). Data for paw withdrawal threshold is presented as mean with s.e.m., and significance was evaluated by Student t-test.

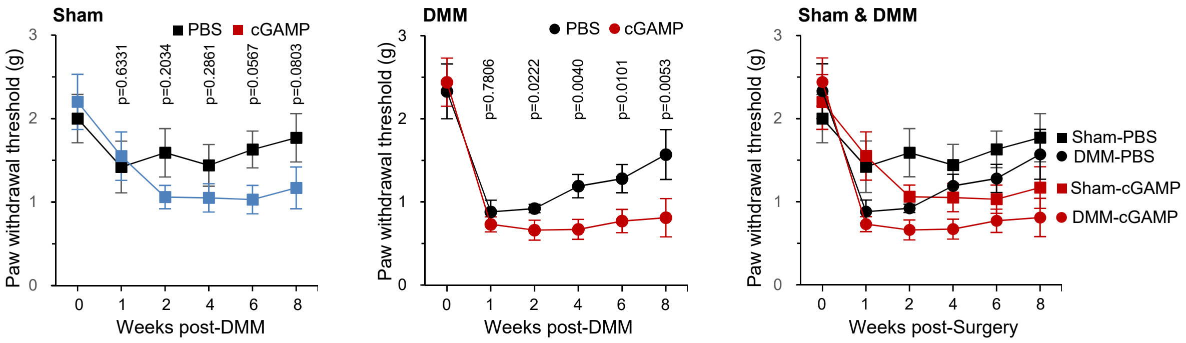

Supplementary Fig. 4. Stimulation of the STING in mouse knee joints exacerbates OA-associated mechanical allodynia. von Frey assays were performed at the indicated weeks after sham operation or DMM surgery in mice IA injected with cGAMP or vehicle (PBS) (n =15 mice per group). Data is presented as mean with s.e.m., and significance was evaluated by Student t-test.

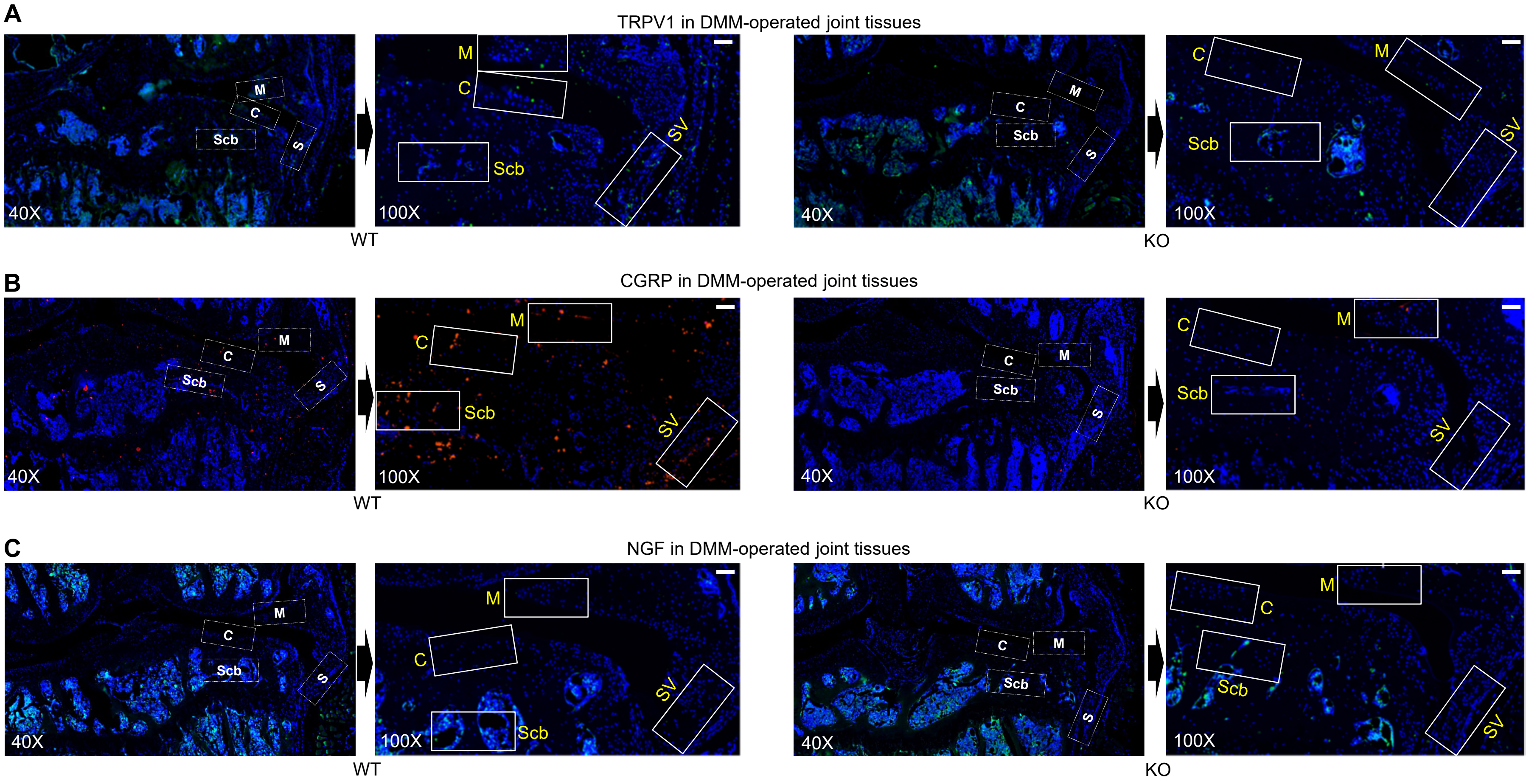

Supplementary Fig. 5. Expression of pain-sensitizing molecules in joint tissues of DMM-operated WT and *Sting1*$^{-/-}$ mice. (A-C) Representative immunostaining images of TRPV1 (A), CGRP (B), and NGF (C) in joint sections of DMM-operated WT and *Sting1*$^{-/-}$ mice (n = 6 mice per group). C: cartilage, M: meniscus, SCB: subchondral bone, SV: synovium. Scale bars: 50 μm.

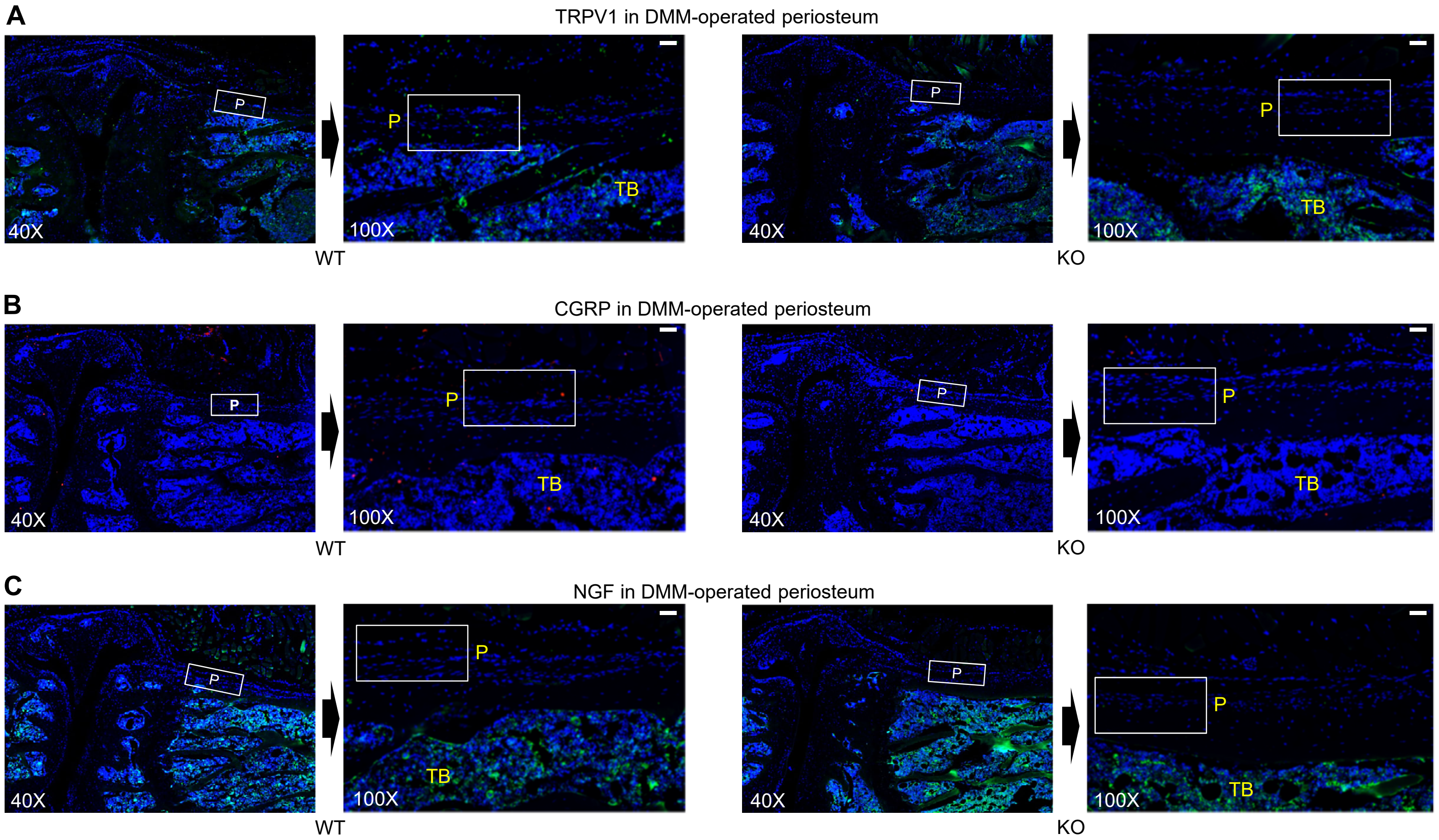

Supplementary Fig. 6. Expression of pain-sensitizing molecules in periosteum of DMM-operated WT and *Sting1*$^{-/-}$ mice. (A-C) Representative immunostaining images of TRPV1 (A), CGRP (B), and NGF (C) in periosteum of DMM-operated WT and *Sting1*$^{-/-}$ mice (n = 6 mice per group). P: periosteum, TB: trabecular bone. Scale bars: 50 µm.

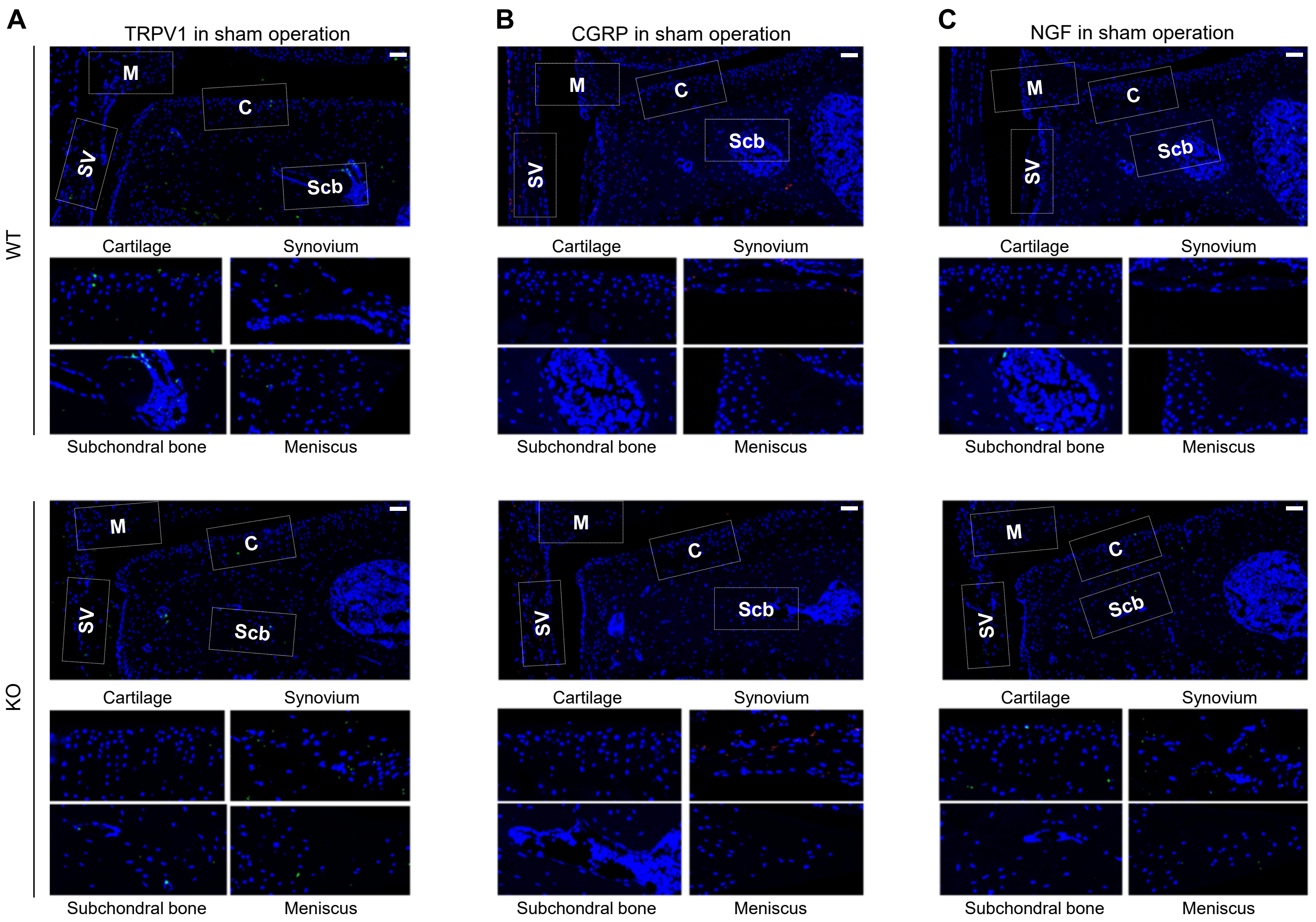

Supplementary Fig. 7. Expression of pain-sensitizing molecules in joint tissue of sham-operated WT and *Sting1*$^{-/-}$ (KO) mice. (A-C) Representative immunostaining images of TRPV1 (A), CGRP (B), and NGF (C) in joint section of sham-operated wild-type (WT) and *Sting1*$^{-/-}$ (KO) mice (n = 6 mice per group). C: cartilage, M: meniscus, Scb: subchondral bone, SV: synovium. Scale bars: 50 µm
